# American Indians and atrial fibrillation

**DOI:** 10.1016/j.hroo.2022.08.010

**Published:** 2022-12-16

**Authors:** José M. Sanchez, Gregory M. Marcus

**Affiliations:** ∗ Department of Cardiology and Electrophysiology, Kaiser Permanente of Colorado, Aurora, Colorado; † Section of Cardiac Electrophysiology, Division of Cardiology, University of California, San Francisco, San Francisco, California

**Keywords:** Atrial fibrillation, American Indians and Alaska Natives, Race and ethnicity, Epidemiology, Indigenous population

## Abstract

The American Indian population is known to experience high rates of cardiovascular disease and have a heightened vulnerability to severe outcomes driven by an overall poor health status and lower access to quality health care. Our group has previously published an analysis demonstrating that American Indians have the highest risk of atrial fibrillation (AF), as well as of AF-related stroke, when compared with other races and ethnicities. Despite this, AF in this population has not been extensively studied and additional publications are scarce. Our review article provides an up-to-date summary of the relevant literature addressing the relationship between race, ethnicity, and AF by focusing on American Indians.

## Graphical abstract

**Figure:**
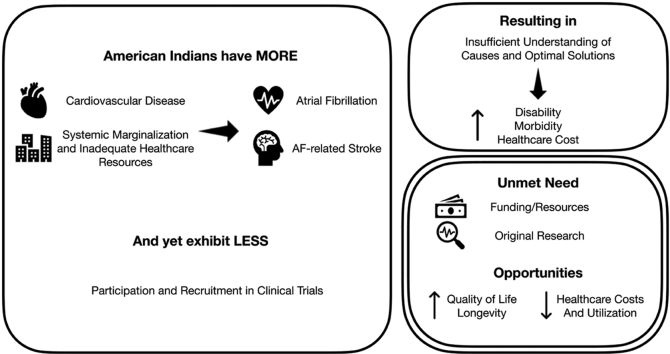

**Key Findings**

▪ American Indians and Alaska Natives experience high rates of cardiovascular disease.
▪ They also have the highest risk of atrial fibrillation (AF) and AF-related stroke compared with other racial and ethnic groups.
▪ We provide an up-to-date summary on the relationship between race, ethnicity, and AF with a focus on American Indians and Alaska Natives.

## Overall health and cardiovascular disease in American Indians and Alaska Natives

It is important to discuss the risks and consequences of atrial fibrillation (AF) in American Indian and Alaska Native populations by first considering the broader context of overall cardiovascular health. In general, American Indians experience particularly high rates of cardiovascular disease (CVD).^1^^,^^2^ American Indians develop CVD at earlier ages than whites, and CVD is the primary cause of death in over one-third of American Indian deaths occurring before the age of 65 years.^1^ This has translated into a lower life expectancy that is 5.5 years less in American Indians than non–American Indians.^3^ A variety of risk factors have been shown to contribute to this heightened rate of CVD.

American Indians have the highest rates of diabetes mellitus, hypertension, chronic kidney disease, smoking, and coronary heart disease in the United States.^1^^,^^2^^,^^4^^,^^5^ Diabetes mellitus is the most important risk factor for CVD in this population, and there is a 3-fold higher age-adjusted prevalence of diabetes compared with whites.^1^ Cigarette smoking, alcohol use, and physical inactivity have also been identified as significant modifiable behaviors strongly linked to overall CVD.^1^ Unlike other groups, smoking rates have not recently declined in American Indians, with estimates that 32% of American Indians smoke compared with 16% in other groups.^6^ Alcohol use is variable but reported to be as high as 60% and highly dependent on geographical location.^7^ Several studies among American Indian and Alaska Native individuals have aimed to investigate the possible genetic role of CVD: it has been observed that heritability patterns of disease exist and contribute to almost 50% of the phenotypes of obesity, dyslipidemia, hypertension, and diabetes mellitus.^8^ A separate study observed that a single set of genes may explain differential risk factors of obesity and low-density lipoprotein size in the American Indian and Alaska Native population.^9^

Social determinants of health and access to quality health care have direct impacts on the overall morbidity and mortality associated with CVD across all populations, phenomena that may be especially pertinent to American Indian individuals. A lower attained level of education has been a reliable predictor of enhanced CVD risk and is related to employment opportunities.^10^ Less than one-quarter of American Indians have an advanced graduate or professional degree, and there are particularly high rates of unemployment.^11^ Poverty rates are highest in American Indians compared with other racial and ethnic minority groups, with certain tribes experiencing nearly triple the national rate.^1^

Finally, American Indians are underinsured, with only 43% having commercial health insurance coverage and 19% having no insurance coverage.^1^ In an effort to ameliorate care gaps in this population, the Indian Health Services (IHS) was established and provides medical services and community programs in primary outpatient and emergency care. IHS, however, provides care to less than one-third of the American Indian and Alaska Native population and, unfortunately, facilities are small and less available in urban centers.^12^ Clinics also lack specialty care and are understaffed by primary care providers with vacancy rates as high as 31%.^12^ This is further exacerbated by low provider salaries when compared with local market rates.^12^ Finally, there are ongoing concerns with overall funding of IHS especially in comparison with other federal health programs. In 2017, IHS per capita spending was 50% less than Medicaid, 62% less than the Veterans Health Administration, and 70% less than Medicare.^12^

Addressing and improving this population’s social determinants of health is complex and requires multifaceted community efforts. There are several IHS- and Centers for Disease Control and Prevention–developed community-based programs that have tried to achieve risk reduction of CVD.^13^^,^^14^ Success of these programs will likely depend on establishing relationships and trust within the community, empowering and supporting tribes; identifying a multidisciplinary team of care managers, health educators, and clinicians; and monitoring progress with an action plan.^1^

## Atrial fibrillation

Despite the fact that American Indians and Alaska Natives generally exhibit substantially increased rates of CVD and established AF risk factors, AF has not been extensively studied in this population. AF is the most commonly encountered cardiac arrhythmia among all Americans and is the second most common cardiovascular condition after hypertension.^15^^,^^16^ In the United States, AF affects 4 million individuals each year, and the prevalence is estimated to increase to at least 12 million by 2030.^17^^,^^18^ The majority of individuals with AF are over the age of 65 years; however, due to rising rates of obesity, diabetes mellitus, and hypertension in young adults, AF is expected to affect more individuals at a younger age.^17^, ^18^, ^19^

AF is associated with significant morbidity and mortality. Individuals with symptomatic AF may experience a myriad of symptoms including palpitations, fatigue, and shortness of breath. Failure to control AF results in decreased quality of life, recurrent hospitalizations, anxiety, and depression.^20^ It is also the most common cause of thromboembolic stroke, increasing the risk for ischemic stroke by 4- to 5-fold.^16^ Noncerebral thromboembolic events have also been associated with AF and can result in myocardial infarction, chronic kidney disease, and dementia.^21^, ^22^, ^23^ Furthermore, AF can cause and worsen heart failure, and it doubles the risk of cardiovascular and all-cause mortality independent of AF risk factors.^24^ Even though the increase risk of death can reflect the fact that AF is often a marker of other underlying concomitant heart and vascular disease, AF likely contributes to adverse outcomes by diminishing cardiac performance due to heart failure and exposing patients to therapies associated with risk.

AF also results in substantial health care utilization and is a considerable economic burden. Patients with AF are 3 times more likely to be hospitalized and 8 times more likely to have multiple cardiovascular hospitalization events and more cardiovascular-related deaths compared with those without AF.^25^ In 2018, there were 5 million office visits, 700,000 emergency department visits, and 472,000 inpatient hospitalizations for AF with a mean length of stay of 3.5 days.^16^ This translates into an estimated $28 billion in health care spending with $20,000–$40,000 spent per patient with AF per year in the United States.^26^^,^^27^

## Risk of developing AF and associated stroke

Race and ethnicity have been shown to have differential effects on AF, affecting not only the prevalence, but also how AF is managed and its associated outcomes. Elucidating these relationships is paramount in understanding AF, particularly among American Indians and Alaska Natives, as racial and ethnic minorities often have worse outcomes, likely rooted in the lower rate of guideline-adherent care in these populations.

The first studies on the topic of race and AF revealed that African Americans have a lower risk of AF compared with non-Hispanic whites despite possessing more AF risk factors.^17^^,^^28^ Additional studies inclusive of other racial and ethnic groups, but not including American Indians and Alaska Natives, subsequently observed that the differences in the prevalence of AF are driven by a higher risk in non-Hispanic whites and not by a lower risk in any particular group.^29^^,^^30^ Of note, these differences are generally dependent on the accuracy of self-reported race and ethnicity. Even though admixed populations exist, this self-identification has been shown to correlate well with genotype-confirmed continental ancestry.^31^ In the case of African Americans, a population known to exhibit varying degrees of European ancestry, an increase in European ancestry was associated with a corresponding increase in incident AF risk.^32^ Whether similar analogous findings apply to admixed populations of American Indians and Alaska Natives remains unknown.

**Figure 1:**
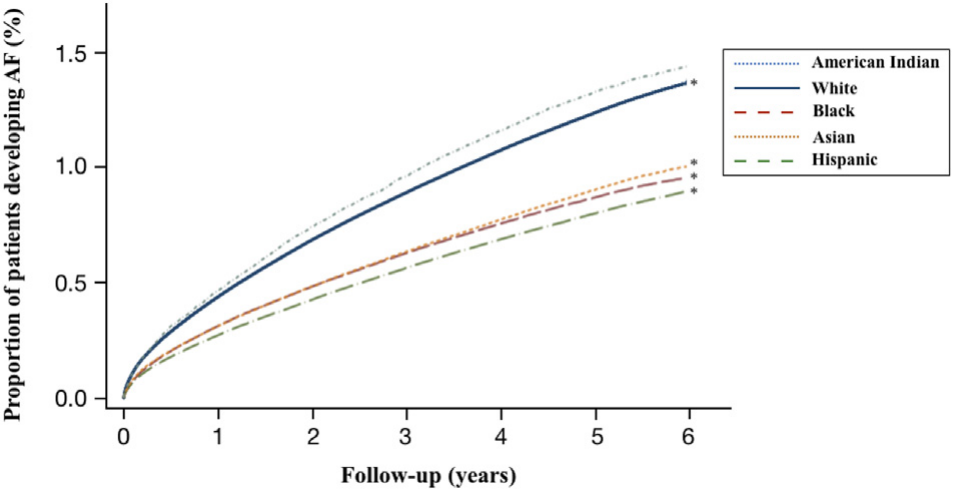
Adjusted Kaplan-Meier curves for incident atrial fibrillation (AF) in American Indian, white, black, Hispanic, and Asian patients. The curves are adjusted for age, sex, income level, insurance payer, hypertension, diabetes, coronary artery disease, heart failure, valvular disease, chronic kidney disease, smoking, sleep apnea, pulmonary disease, alcohol use, and number of healthcare encounters. ∗Comparison between American Indians to each individual race and ethnicity, *P* value <.0001. Reprinted with permission from Lippincott Williams & Wilkins.^34^

Studies examining the risk of AF among American Indians compared with other racial and ethnic groups are relatively scarce. One investigation found that American Indians had a high prevalence of AF compared with other minority groups, but with a similar prevalence to whites.^33^ However, this was a cross-sectional study limited only to male veterans in the United States. Our group subsequently utilized 3 Healthcare Cost and Utilization Project databases to identify 101,848 American Indians in California who received care in an emergency department, inpatient hospital unit, or ambulatory surgery setting over a 6-year period.^34^ In this longitudinal study, we found that American Indians exhibited a higher risk of incident AF compared with other races and ethnicities (Figure 1). These results persisted after multivariable adjustment as well as after sensitivity analyses. While these findings are representative of the AF risk accrued by American Indians and Alaska Natives, other indigenous populations outside of the United States may have a similar propensity to AF; data, however, are sparse. Aboriginal and Maori individuals in Australia and New Zealand, respectively, have been found to develop AF 10 years earlier than whites living in the same country.^35^^,^^36^ Metis individuals in Canada have also been shown to have a higher AF prevalence than others.^37^ While the indigenous populations outside of the United States are quite distinct from American Indians, they collectively share similar historical experiences, socioeconomic disadvantages, and overall health status.^37^ Furthermore, we speculate that this heightened risk of AF seen across geographically distinct indigenous populations is likely attributable to an unidentified characteristic, including possible genetic or environmental factors.

Identifying a biologic mechanism that underlies these observed differential risks in developing AF would help uncover processes responsible for the etiology of AF. Our group has shown that there are racial variations in the size of the left atrium and risk of conduction disease, and differences may translate into a higher frequency of premature atrial contractions, which have been shown to be an independent predictor of AF.^38^, ^39^, ^40^, ^41^, ^42^ Furthermore, certain biomarkers have a potential role in the risk of AF, and some evidence suggests that almost half of the racial difference in AF may be mediated by increased levels of inflammatory markers.^43^ Taken together, these findings suggest that certain racial and ethnic groups may be more prone to developing cardiac ectopy, fibrosis, or both, as a pathway to the development of AF. These studies, however, have not traditionally included American Indian and Alaska Native individuals, and the mechanisms by which this population may experience a particularly heightened risk of AF have not yet been determined, representing an important novel field for research.

**Figure 2:**
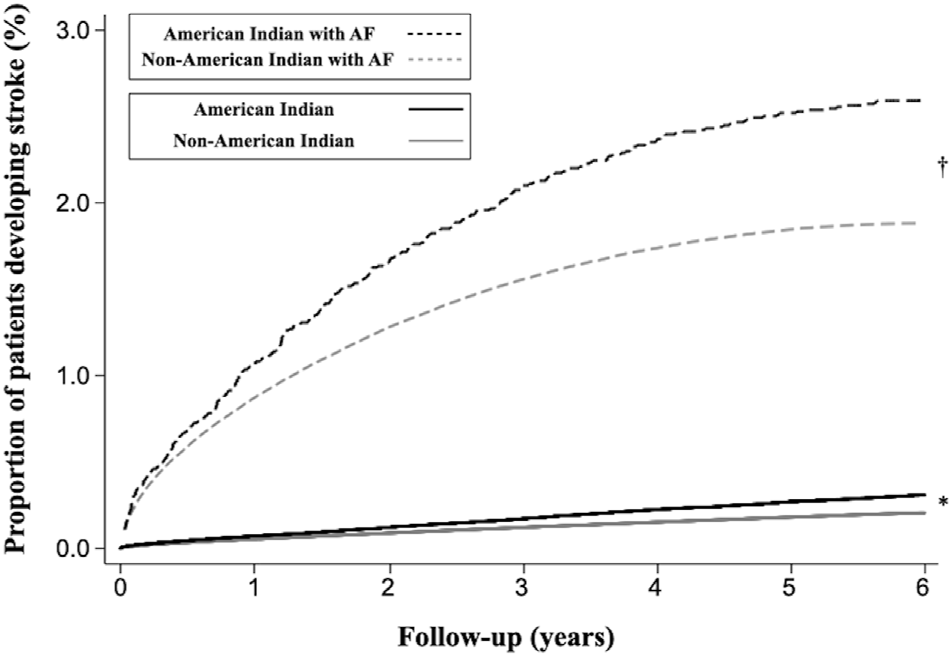
Adjusted Kaplan-Meier curves for incident nonhemorrhagic stroke in American Indian and non–American Indian patients with and without atrial fibrillation (AF). The curves are adjusted for age, sex, income level, insurance payer, hypertension, diabetes, coronary artery disease, congestive heart failure, cardiac surgery, valvular heart disease, chronic kidney disease, smoking, obstructive sleep apnea, pulmonary disease, and alcohol use. ∗Comparison between American Indians to non–American Indians, *P* value <.0001. †Comparison between American Indians with AF and non–American Indians with AF, *P* value <.0001. Reprinted with permission from John Wiley & Sons Inc.^47^

In addition to the effects on the prevalence of AF, race and ethnicity are also important factors in determining the risk of AF-related stroke. In populations with AF, black individuals have a particularly high risk of nonhemorrhagic stroke, followed by Hispanic and then white individuals.^44^^,^^45^ This relationship between race, ethnicity, and AF-related stroke plays an important role in clinical practice, in which inclusion of the black race to the CHA_2_DS_2_-VASc (congestive heart failure, hypertension, age ≥75 years, diabetes mellitus, prior stroke or transient ischemic attack or thromboembolism, vascular disease, age 65–74 years, sex category) scoring system has been shown to significantly improve the prediction of stroke among those newly diagnosed with AF.^45^ Unfortunately, inclusion of American Indians and Alaska Natives in similar studies exploring the effect of AF on stroke risk in this population is even more rare. There are observational studies that have demonstrated a high incidence of overall stroke in American Indians compared with white, black, and Hispanic individuals,^1^^,^^46^ but the influence of AF on the stroke risk compared with other groups was not investigated. Using the same California Healthcare Cost and Utilization Project databases that were utilized in our incident AF analysis,^34^ we subsequently confirmed that American Indians are at an elevated risk of nonhemorrhagic stroke compared with other races and ethnicities.^47^ When individuals with AF were included, we discovered that American Indians with AF exhibited the highest risk of nonhemorrhagic stroke compared with other minority groups with AF (Figure 2).^47^

The basis for the heightened risk of stroke in American Indians with AF remains unknown and is another related area deserving of future research. Interestingly, it has been demonstrated that the risk of stroke in some minority groups persists despite adjustment for anticoagulation status.^44^ Racial and ethnic groups may more often experience poor control of hypertension, diabetes mellitus, and congestive heart failure, each of which may augment nontraditional risk factors or result in systemic inflammation or other processes leading to an increased risk of stroke.^48^^,^^49^ Finally, a strong emphasis on the effect of socioeconomic status has been proposed as well as a differential impact of certain stroke risk factors across racial and ethnic groups.^50^

## Treatment and associated outcomes among individuals with AF

Disparities in the management and treatment of AF are pervasive, and because racial and ethnic groups, in particular American Indians, have been shown to have reduced access to health care, significant gaps in AF treatment and associated outcomes are expected. In general, the management of AF is aimed at controlling symptoms and preventing stroke. Even though rate control is a reasonable approach for those with symptomatic AF, the frequency of rhythm control with antiarrhythmic drug therapy or catheter ablation is increasing among individuals with AF.^51^ Furthermore, rhythm control has been shown to improve quality of life and halt accompanying structural and electrical adverse remodeling, and has been associated with improved cardiovascular outcomes.^15^^,^^52^^,^^53^ Catheter ablation decreases mortality and heart failure hospitalizations in those with heart failure with reduced ejection fraction and AF,^54^^,^^55^ and current guidelines recommend catheter ablation as a first-line treatment for symptomatic AF.^15^

Despite ongoing advancements in rhythm control and its proven favorable clinical outcomes, access to these cardiovascular therapies among minority groups and those from lower socioeconomic class remains limited.^56^ Individuals who undergo AF ablation are predominantly white and male.^57^ In a population of individuals with commercial insurance, blacks as well as Hispanics with a lower median household income were independently less likely to receive catheter ablation and rhythm control strategies, in general.^51^ Among American Indians and Alaska Natives, a study utilizing the National Cardiovascular Data Registry Practice Innovation and Clinical Excellence (PINNACLE) registry, the largest cardiovascular outpatient registry in the United States, discovered that American Indians were significantly less likely to be treated with rhythm control strategies compared with non–American Indians.^58^

Finally, prevention of systemic thromboembolic events and AF-related stroke is paramount in the treatment of AF. Long-term oral anticoagulation for nonvalvular AF reduces the risk of stroke by up to 70% and is the standard of care in patients with moderate-to-severe stroke risk.^59^^,^^60^ While this risk reduction has traditionally been achieved with warfarin, direct oral anticoagulants have demonstrated superior clinical outcomes, cost effectiveness, safety, and adherence compared with warfarin.^61^^,^^62^ National guidelines have now endorsed direct oral anticoagulants as first-line anticoagulation therapy for individuals with an elevated risk of AF-related stroke.^63^ Despite this, racial and ethnic disparities in the use of oral anticoagulation exist. Prior studies have shown that minority groups with AF were less likely than white individuals to be treated with any form of oral anticoagulation.^64^^,^^65^ Specifically among American Indian and Alaska Native individuals, those with AF were less likely to be treated with oral anticoagulation compared with non–American Indians with AF.^58^ The extent of these observed disparities is amplified by the fact that various racial and ethnic groups have higher rates of AF-related stroke and mortality than white individuals.^45^^,^^47^^,^^66^^,^^67^

## Conclusion and future directions

AF and AF-related stroke disproportionately more often affect American Indians and Alaska Natives. A multifaceted approach is needed to understand the racial variation in AF development and management; however, this has been hampered by under-recruitment of racial minorities in both prospective observational studies and randomized clinical trials. Community health measures aimed at risk factor modulation and improved AF management once a diagnosis has been established, including guideline-adherent anticoagulation prescribing patterns, are pivotal. For example, given the particularly high-risk of AF as well as AF-related stroke in American Indians, screening for AF in this population may be reasonable.^68^ Remedies to improve equal delivery of high-quality care are complex, and an improved public health model forged to address the challenges unique to American Indians and Alaska Natives would help decrease the burden and complications of AF in this traditionally underserved group.
